# Will It Be Possible in the Future to Target Innate Immunity Against *Staphylococcus aureus* Using Resident Microbiota and Plant Extracts?

**DOI:** 10.1155/jimr/3556897

**Published:** 2026-08-29

**Authors:** Urszula Wójcik-Bojek, Joanna Rywaniak, Beata Sadowska

**Affiliations:** ^1^ Department of Immunology and Infectious Biology, Faculty of Biology and Environmental Protection, University of Lodz, Lodz, Poland, uni.lodz.pl

**Keywords:** immunomodulation, innate immunity, macrophages, plant extracts, staphylococci, *Viburnum opulus*

## Abstract

*Staphylococcus aureus* remains a major cause of hospital‐ and community‐acquired infections, successfully evading host defense mechanisms and escaping proposed vaccination strategies. The effective response of professional phagocytes is crucial in limiting the spread, tissue invasion, and subsequent infection by *S. aureus*. However, direct stimulation of innate immunity with *S. aureus* antigens is too risky due to their strong proinflammatory properties. Therefore, this study aimed to test the hypothesis that plant extracts trigger the gentle release of staphylococcal bioactive components that can modulate innate immunity. This strategy was tested in vitro; however, it may have future applications by employing the resident microbiota and dietary supplements containing plant extracts. In this study, the THP‐1‐derived macrophages were exposed to the supernatants of planktonic and biofilm *S. aureus* cultures pretreated with *Viburnum opulus* L. bark and fruit extracts. Vancomycin (VAN) and chlorogenic acid (ChA), used at subinhibitory concentrations, were included for comparison regarding the release of *S. aureus* active components. The cells were also exposed to purified bacterial cell wall components. Various elements of the immune response were assessed, including surface expression of cluster of differentiation (CD11c, CD206) via flow cytometry, cytokine production (tumor necrosis factor‐alpha [TNF‐α] and interleukin 10 [IL‐10]) via ELISA, and *S. aureus* phagocytosis and intracellular killing using fluorescein isothiocyanate (FITC)‐labeled bacteria and Alamar Blue staining. It was demonstrated that both supernatants from planktonic and biofilm *S. aureus* cultures pre‐exposed to *V. opulus* extracts, as well as the bacterial cell wall components themselves, significantly increased TNF‐α and IL‐10 production by THP‐1‐derived macrophages and visibly reduced intracellular multiplication of engulfed *S. aureus*. The lack of changes in the surface CD marker expression may be a limitation of the model used (phorbol‐12‐myristate‐13‐acetate [PMA]‐differentiated THP‐1) rather than the phenomenon itself, that leads to a “ceiling effect”. To sum up, the study presents *V. opulus* extracts as the triggers to release bioactive components from *S. aureus* cells, able to target innate immunity against staphylococci, which may be used in the future.

## 1. Introduction

Knowledge of *Staphylococcus aureus* virulence factors, metabolites, physiology, behavior, and communities formed, as well as evasion of the immune system and pathological changes caused, is extensive [1–8]. Nevertheless, these bacteria are still one of the most common human and animal pathogens, causing a wide spectrum of infections ranging from moderately severe skin and soft tissue infections, organ abscesses, biomaterial/biofilm‐associated infection (BAI), and foodborne infections/poisoning to life‐threatening bacteremia, bone, joint, and bone marrow infections, hospital‐acquired pneumonia, infective endocarditis, sepsis, or toxic shock syndrome [4, 9–11]. Due to the widespread prevalence of *S. aureus* drug‐resistant strains (mainly methicillin‐resistant *S. aureus* [MRSA]), the excellent ability of these bacteria to avoid host immune defense mechanisms, and the participation of biofilms in the development of pathological lesions, staphylococcal infections are particularly problematic. According to the World Health Organization (WHO), MRSA and vancomycin (VAN)‐resistant *S. aureus* (VRSA) are high‐priority pathogens for the research of new antibiotics [12]. Because of this, alternative therapeutic solutions in the fight against staphylococcal infections are urgently needed. The dynamics of *S. aureus* infections depend primarily on the activity of innate immune cells. A fast and efficacious response of professional phagocytes (macrophages and neutrophils) can hold staphylococci at the site of entry and control their dissemination [13–15]. It has been shown in animal models that quantitative and functional depletion of macrophages leads to an increase in the *S. aureus* load, animal morbidity, and mortality [9, 14, 16]. Moreover, Chan et al. [9] demonstrated that priming of animals by prior *S. aureus* infection reduced skin lesion severity and MRSA burden, inhibiting the late dissemination of bacteria. Innate immune response during *S. aureus* recurrent infection in mice (increased number of M1 macrophages in the abscesses and total macrophages in lymph nodes and enhanced efficacy of bone marrow‐derived macrophages against staphylococci) indicates that protective immunity against staphylococci involves specific memory conferred by macrophages [9]. Thus, research on immunomodulation and training of innate immune cells may be important in the context of alternative methods for preventing and treating staphylococcal infections.

Numerous studies are currently conducted to search for substances with immunomodulatory properties that could be used, for example, as vaccine adjuvants, medicines, or supplements to boost host immunity. Among them are microbial and plant‐origin components and products, including plant extracts, essential oils, or plant exosome‐like nanovesicles. Plant extracts themselves possess many beneficial activities, such as antioxidative, antiproliferative, anticancer, antimicrobial, antiallergic, diuretic, topically anesthetic, and immunomodulatory effects [17–22]. Previously, we extensively studied antistaphylococcal properties of biochemically well‐characterized (HPLC/UPLC–QTOF–MS) original acetonic, ethanolic, and water extracts from fruits and bark of *Viburnum opulus* L. We have previously demonstrated that *V. opulus* extracts strongly inhibited *S. aureus* sortase A (SrtA) activity and staphylococcal protein A (SpA) surface exposure, limited biofilm formation by staphylococci, and also caused modifications of *S. aureus* cell membrane [23]. Such an effect on the staphylococcal membrane and cell wall gave us the idea of using these extracts as agents that gently release immunomodulators from the cells of these bacteria. It is known that many components of microbial cells (e.g., peptidoglycan [PG], lipoteichoic acids [LTAs], lipopolysaccharide [LPS], β‐glucan [BG]) exert an immunomodulatory effect [24–27]. We demonstrated that some biologically active staphylococcal components/products (LTA, staphylokinase [SAK], and hemolysin alpha [HLA]) were released from both planktonic and biofilm *S. aureus* cultures [28]. Moreover, surface‐acting preparations, including such antibiotics as β‐lactams or glycopeptides, stimulate the release of such components from microbial cells [29–32]. This may, in the future, open up the possibility of applying the resident microbiota as a source of immunomodulators. However, due to the problem of drug resistance among microorganisms, as well as the risk of dysbiosis and massive release of proinflammatory factors possibly leading to sepsis and septic shock [33–37], the usage of antibiotics for preventive purposes is limited. Instead here, we checked the applicability of plant‐origin natural extracts to the gentle and long‐lasting release of biologically active components from the cells of staphylococci. We wanted to explore the indirect immunomodulatory potential of plant extracts and the possibility of using, in the future, the resident microbiota as a source of immunomodulators to prevent staphylococcal infections.

In this study, biochemically well‐characterized ethanolic (e) and water (w) extracts from fruits and bark of *V. opulus* L. (*V. opulus* fruit [VF] and *V. opulus* bark [VB], respectively) were used as the triggers to release biologically active components from *S. aureus* ATCC 43300 (MRSA) growing in planktonic and biofilm cultures. Chlorogenic acid (ChA) as a reference compound detected in all tested *V. opulus* extracts, and VAN at half of the minimum inhibitory concentration (MIC) to obtain comparative *S. aureus* culture supernatants was used. Subsequently, the THP‐1‐derived macrophages were exposed to the *S. aureus* culture supernatants and cell activation parameters, including selected cluster of differentiation markers (CD11c as a marker of M1 and CD206 as a marker of M2) surface exposure (flow cytometry; FACS), cytokines (tumor necrosis factor‐alpha [TNF‐α] and interleukin 10 [IL‐10]) production (ELISA), *S. aureus* phagocytosis, and intracellular killing (fluorescence of fluorescein isothiocyanate [FITC]‐labeled bacteria; Alamar Blue staining) were assessed. For comparison, the cells were also exposed to commercially available purified components of bacteria: *S. aureus* PG and SpA, as well as *E. coli* LPS.

## 2. Materials and Methods

### 2.1. Plant Material Preparation and Chemical Analysis

Commercially available dried bark and fruits of *V. opulus* L. were purchased from “FarmVit” (Szczecin, Poland) and “NaturaWita Ltd.” (Kopernia, Poland), respectively. Preparation, chemical analysis, and composition of the extracts were detailed and described in our previous work [23]. Finally, VBw (VB water) extract, VFw (VF water) extract, and VFe (*V. opulus* fruit ethanolic) extract were used in this study.

### 2.2. Preparation of Stock Solutions of the Extracts and Cell Wall Components

Stock solutions of tested *V. opulus* extracts (10 mg/mL) were prepared in sterile‐filtered water and kept frozen at −20°C. Further dilutions of each stock were prepared in a medium suitable for the type of experiment. The reference compound—ChA (SERVA Feinbiochemica, Germany)—was dissolved in 50% ethanol (Et‐OH) to obtain a stock solution (10 mg/mL), which was kept frozen at −20°C and diluted in an appropriate medium for each experiment (the final Et‐OH concentration in bacterial culture exposed to ChA reached 0.5%). PG from *S. aureus* (Sigma–Aldrich, USA) and SpA (Pharmacia Fine Chemicals, Sweden) were dissolved in sterile, pyrogen‐free water for injection (Sigma–Aldrich, USA) at 1 mg/mL and 2.5 mg/mL, respectively. PG and SpA stock solutions were frozen at −20°C and diluted in an appropriate medium for each experiment.

### 2.3. *S. aureus* Culture Conditions

The stock suspension of *S. aureus* ATCC 43300 (reference MRSA strain) in tryptic soy broth (TSB; BTL Sp. z o.o., Poland) containing 15% glycerol (POCH, Poland) kept frozen at −80°C was grown in tryptic soy agar (TSA; BTL Sp. z o.o., Poland) for 24 h at 37°C. Then, *S. aureus* cultures in TSB with 0.25% glucose (TSB/Glu) and/or suspensions in phosphate‐buffered saline (PBS, Biowest, France) were freshly prepared before setting up the experiments. The suspension density was adjusted to the appropriate value for the given test.

### 2.4. Assessment of MIC and Minimum Bactericidal Concentration (MBC) of VAN

The MIC of VAN (Sigma–Aldrich, USA) tested at a final concentration range of 4–0.06 µg/mL against *S. aureus* ATCC 43300 was determined with a microdilution broth assay according to the EUCAST recommendations [38]. *S. aureus* suspension (10^5^ CFU/mL) was exposed to the series of VAN dilutions for 24 h at 37°C. Bacteria in the medium alone were prepared as a growth control. The MIC was defined as the lowest antibiotic concentration inhibiting the visible growth of bacteria during coincubation. To evaluate the MBC value, 10 µL from the wells marked as the MIC and two wells with higher antibiotic concentrations were subcultured in TSA for 24 h at 37°C. The MBC refers to the lowest antibiotic concentration required to kill 99.9% of bacteria. A concentration of ½ MIC VAN was used for further experiments.

### 2.5. Exposure of *S. aureus* Planktonic and Biofilm Cultures to Plant Extracts, ChA or VAN, and Collection of the Supernatants

To collect planktonic supernatants, *S. aureus* ATCC 43300 suspension in TSB/Glu (OD_535_ = 0.5) was exposed to *V. opulus* extracts (100 µg/mL), ChA (100 µg/mL), or VAN (½ MIC) for 24 h at 37°C on an orbital shaker type TR‐250/CH‐4103 (Bottmingen, Switzerland) in a total sample volume of 1 mL. For biofilm supernatants, *S. aureus* ATCC 43300 suspension in TSB/Glu (OD_535_ = 1.0) was first plated into the wells (1 mL/well) of 24‐well polystyrene culture microtiter plates (Corning Incorporated, USA) for 24 h at 37°C to develop a biofilm. Two types of control were set up simultaneously: bacteria in TSB/Glu alone (control K1) and in TSB/Glu with 0.5% Et‐OH (control K2) to exclude the impact of Et‐OH being a solvent for ChA. After exposure, planktonic bacteria were centrifuged at 3000 rpm/10 min. The supernatants were collected. The wells with the created *S. aureus* biofilm were gently washed with PBS, and prepared stimulators were added for the next 24 h at 37°C. Then, the plates were centrifuged at 3000 rpm/10 min. The supernatants were collected. Planktonic and biofilm supernatants were filtered using sterile, syringe‐driven filter units (0.22 μm pore size) with a low‐protein binding Durapore PVDF membrane (Merck Millipore Ltd., Ireland) and stored at −80°C for further experiments. Supernatants were prepared from five independent experiments.

### 2.6. Assessment of PG and SpA Release to the Supernatants Using ELISA

To assess the presence of PG and SpA in the collected *S. aureus* culture supernatants, commercial ELISA tests (Mybiosource, USA) were used according to the manufacturer’s instructions. The appropriate controls were used: supernatant from bacteria in TSB/Glu alone (K1) as the control for all samples except that exposed to ChA and supernatant from bacteria in TSB/Glu with 0.5% Et‐OH (K2) as the control for the ChA sample only. Two independent experiments were performed using the supernatants from four preparative experiments. The results are presented as the mean concentration (ng/mL) ± SD (*n* = 4).

### 2.7. THP‐1 Cell Culture Conditions and Differentiation to the Macrophages

Human monocyte line THP‐1 (ATCC TIB‐202) was purchased from LGC Standards Sp. z o.o. (Poland) and cultured according to manufacturer’s instruction in RPMI‐1640 with L‐glutamine medium (Sigma Life Science, USA) supplemented with 10% (v/v) fetal bovine serum (FBS; Biowest, France), 1% (v/v) penicillin‐streptomycin solution (P/S; Biowest, France), and 0.05 mM 2‐mercaptoethanol (Bio‐Rad, USA) at 37°C in a humidified atmosphere of 5% CO_2_. To differentiate THP‐1 cells into macrophages, phorbol‐12‐myristate‐13‐acetate (PMA; InvivoGen, USA) at a final concentration of 200 nM was added to 5 × 10^5^ cells/mL suspension, and the cells were seeded into the 24‐well (2.5 × 10^5^ cells/well) or 96‐well (1 × 10^5^ cells/well) microtiter plates (Corning Incorporated, Corning, USA) for 72 h at 37°C/5% CO_2_. Then, the medium with PMA was removed, and the cells were washed once with PBS without calcium and magnesium ions (Biowest, France) or Hanks Balanced Salt Solution with HEPES (HHBS; Sigma Life Science, USA) when the macrophage phagocytic activity was tested. The complete cell culture medium was then added, and the cells rested for a further 72 h at 37°C/5% CO_2_ before starting the experiments.

### 2.8. Macrophage Exposure to Staphylococcal Culture Supernatants and Pure Cell Wall Components

Upon resting, THP‐1‐derived macrophages were cultured for 24 h at 37°C/5% CO_2_ in cell culture medium containing *S. aureus* ATCC 43300 planktonic and biofilm‐tested and comparative supernatants (10%, v/v), PG (10 and 20 µg/mL), and SpA (10 and 100 µg/mL). Appropriate control cells (K) in cell culture medium alone (control for the cells exposed to bacterial cell wall components) and cell culture medium containing 10% (v/v) TSB/Glu (control for the cells exposed to the supernatants) were set up at the same time. After exposure, the cell‐free supernatants were collected and stored at −80°C for testing of TNF‐α and IL‐10 presence, whereas the cells were used for further analyses.

### 2.9. Assessment of TNF‐α and IL‐10 Production Using ELISA

The assessment of TNF‐α and IL‐10 presence in cell‐free supernatants was performed using commercially available DuoSet ELISA kits (R&D Systems, Minneapolis, MN, USA) according to the manufacturer’s instructions. Necessary buffers were prepared separately using potassium chloride (Standard Sp. z o.o., Poland), sodium chloride (Standard Sp. z o.o., Poland), disodium hydrogen phosphate (POCH, Poland), potassium dihydrogen phosphate (POCH, Poland), Tween 20 (Merck, Germany), bovine serum albumin (BSA; Sigma Life Science, USA), and sodium dodecyl sulfate (SDS; Sigma–Aldrich, USA). Ready to use 2,2^′^‐azinobis‐(3‐ethylbenzothiazoline‐6‐sulfonic acid) (ABTS; Sigma–Aldrich, USA) was applied for color development. Four separate experiments were run in duplicate using the supernatants from four preparative experiments.

### 2.10. Evaluation of the CD Marker Expression on Postexposed THP‐1‐Derived Macrophages Using Flow Cytometry

THP‐1‐derived macrophages exposed (24 h) to tested and comparative supernatants and staphylococcal cell wall components, as well as nonexposed control cells (K), as described above, were centrifuged (1200 rpm/5 min) and gently washed once with 15 mM EDTA (POCH, Poland) solution in PBS. The cells were then detached with a 15 mM EDTA/0.5% BSA solution in PBS (30 min, on ice) and transferred to Eppendorf tubes for centrifugation (3000 rpm/5 min). Obtained cell pellets were resuspended in 200 µL of staining buffer (0.5% BSA/0.1% sodium azide in PBS), and 50 µL aliquots were transferred to cytometric tubes and stained with a mixture of fluorochrome‐labeled antibodies: PerCP‐Cy5.5 Mouse Anti‐Human CD45 antibodies, V450 Mouse Anti‐Human CD11c antibodies, and FITC Mouse Anti‐Human CD206 antibodies (Becton Dickinson, USA). Simultaneously, FMO (fluorescence minus one) controls were prepared. All samples were incubated for 30 min on ice, then 1 mL of staining buffer was added, and the samples were centrifuged (3000 rpm/5 min). Finally, cell pellets were resuspended in a staining buffer and measured on a BD LSRII flow cytometer (Becton Dickinson, USA). The cytometer settings and compensation adjustments were made using a BD CompBeads Compensation Particles Set (Becton Dickinson, USA) and fluorochrome‐labeled antibodies, which were used in the experiments. CD11c and CD206 surface expression were measured on CD45‐positive cells using BD FACSDIVA software (Becton Dickinson, USA). The cells were distinguished from debris based on the light scatter profile (FSC‐A vs. SSC‐A). Then, the singlet gating was set (FSC‐H vs. FSC‐A) to discriminate cell doublets. Macrophages were gated based on the expression of CD45 (CD45^+^ objects). The expression of selected receptors (CD11c and CD206) on the macrophages (CD45^+^ objects) was gated against FMO. The experiment was repeated four times using the supernatants from four preparative experiments.

### 2.11. Assessment of the Engulfment of *S. aureus* by Postexposed THP‐1‐Derived Macrophages

THP‐1‐derived macrophages exposed (24 h) to tested and comparative supernatants and staphylococcal cell wall components, as well as nonexposed control cells (K), as described above, were washed with HHBS. *S. aureus* ATCC 43300 suspension in PBS (OD_535_ = 1.0) was centrifuged (3000 rpm/10 min), and 1 mg/mL FITC (Sigma–Aldrich, USA) in PBS was added to the bacterial pellet in a volume equal to the initial volume of staphylococcal suspension to prepare FITC‐labeled bacteria. Bacteria with FITC were incubated for 20 min on an orbital shaker, in the dark, at room temperature, and then washed three times with 4% BSA in PBS, centrifuging each time under the conditions as above. Finally, the pellet of FITC‐labeled bacteria was resuspended in the cell culture medium without antibiotics and added to the cells at an MOI of 10 for 1 h at 37°C/5% CO_2_. Simultaneously, the macrophage cultures without bacteria were set up to evaluate the autofluorescence of the cells. Free‐floating bacteria were then removed by gently washing the cell monolayer with HHBS, and the fluorescence of bacteria adhered to the cells (F1) was measured at 485_ex_/535_em_ nm using SpectraMax i3 (Molecular Devices, USA) in the Laboratory of Microscopic Imaging and Specialized Biological Techniques at the Faculty of Biology and Environmental Protection, University of Lodz. Next, 0.2% Triton X‐100 (Sigma–Aldrich, USA) in PBS was added for 10 min at room temperature on an orbital shaker to lyse the cells and release the engulfed staphylococci. The fluorescence of the samples—bacteria adhered to the cells and engulfed (F2)—was measured again as above. Calculations: first, the mean value of the cell autofluorescence was subtracted from F1 and F2 values, then a difference between F2 and F1 for each sample was used to calculate a percentage of *S. aureus* phagocytosis (engulfment) compared to the appropriate control considered as 100% of phagocytosis. The experiment was repeated five times in quadruplicate using the supernatants from five preparative experiments.

### 2.12. Assessment of the Intracellular Killing of Engulfed *S. aureus* by Postexposed THP‐1‐Derived Macrophages

THP‐1‐derived macrophages exposed (24 h) to tested and comparative supernatants and staphylococcal cell wall components, as well as nonexposed control cells (K), as described above, were washed with HHBS. *S. aureus* ATCC 43300 suspension in PBS (OD_535_ = 1.0) was diluted 10 times in PBS, centrifuged (3000 rpm/10 min), resuspended in the cell culture medium without antibiotics in a volume equal to the initial volume of staphylococcal suspension and added to the cells at an MOI of 10 for 1 h at 37°C/5% CO_2_. Simultaneously, the macrophage cultures without bacteria were set up to evaluate the autofluorescence of the cells. Free‐floating bacteria were then removed, and the cells were treated with 50 µg/mL gentamycin (Sigma–Aldrich, USA) in the cell culture medium for 1 h at 37°C/5% CO_2_. Then, the cells were washed once with HHBS. To assess initial *S. aureus* engulfment, the cells were immediately lysed with 0.2% Triton X‐100 in PBS (10 min at room temperature on an orbital shaker), and released bacteria were stained with Alamar Blue Cell Viability Reagent according to the manufacturer’s instructions (Invitrogen, USA). While cell culture medium was added (200 μL/well) to the cells intended for testing of *S. aureus* intracellular killing, and the samples were incubated further for 24 h at 37°C/5% CO_2_. Next, these cells were treated analogously. The fluorescence (F) of the samples was measured at 550_ex_/585_em_ nm using a SpectraMax i3. Calculations: first, the mean value of the cell autofluorescence was subtracted from all *F* values of the samples (at 1 and 24 h). Then, a percentage of *S. aureus* intracellular survival was calculated for each sample by comparing *F* at 24 h to *F* at 1 h, considered as 100% of engulfed bacteria. The experiment was repeated four times in quadruplicate using the supernatants from four preparative experiments.

### 2.13. Statistics

The results were presented as the median and interquartile range (IQR). The normality of the data distribution was assessed prior to statistical testing. For dependent data, differences between groups were assessed using the Wilcoxon signed‐rank test for two‐group comparisons and the Friedman ANOVA test, followed by the Dunn’s post hoc test for multiple comparisons. Statistical significance was set at *p* < 0.05. Results were visualized using box plots, which display the median, IQR, minimum, and maximum. Statistical analysis was performed using Statistical software Version 13.3 (StatSoft, Tulsa, OK, USA).

## 3. Results and Discussion

Several studies point to professional phagocytes (macrophages and neutrophils) as crucial to stop *S. aureus* multiplication, tissue invasion, and the escalation of infection [2, 9, 14, 39–41]. Recently, the ability of macrophages to memorize microbial fingerprinting, so‐called trained innate immunity, has been studied [9, 14, 16, 42, 43]. It was demonstrated that a super low dose of LPS (5 pg/mL) itself did not trigger noticeable macrophage activation but caused a distinct effect by priming cells for more robust expression of proinflammatory mediators in response to a second high‐dose LPS challenge [44]. Staphylococcal molecular patterns such as PG or lipoproteins are recognized by innate immune cells via toll‐like (TLR), NOD‐like (NLR), and C‐type lectin (CLR) receptors, which trigger inflammatory pathways responsible for bacterial clearance [2, 39]. The direct administration of staphylococcal components into the body is not possible due to their strong proinflammatory properties and potential adverse effects. However, when released in sufficient quantities, they could serve as antigens for modulating and training host innate immune cells. Thus, we asked whether plant extracts could be used to gently release bacterial components from commensal staphylococci, which ultimately enable modulation and priming of innate immunity against *S. aureus*. Previously, we demonstrated that *V. opulus* extracts caused modifications in the lipid composition of *S. aureus* cell membranes [23]. Thus, a release of cell wall components from these bacteria by *V. opulus* extracts can be expected, similar to the effects of subinhibitory concentrations (sub‐MICs) of antibiotics [24, 29, 32, 45–47]. To check such a possibility and to assess the immunomodulatory potential of such microbial component mixtures, we tested THP‐1‐derived macrophage response and anti‐infectious functions after exposure to *S. aureus* ATCC 43300 (MRSA) planktonic and biofilm culture supernatants pretreated with *V. opulus* extracts.

### 3.1. *V. opulus* Extracts Promote the Release of *S. aureus* Cell Wall Components

To analyze the effect of *V. opulus* extracts used at subinhibitory concentrations on the release from *S. aureus* cells of the molecules capable of modulating the activity of immune cells, the contents of such relevant cell wall components as PG and SpA were measured in bacterial culture supernatants. PG is essential for almost all bacteria but also strongly affects diverse processes in the host body after release from commensal bacteria, mainly those residing in the gastrointestinal tract. According to research focusing on PG distribution, PG fragments are able to cross the intestinal barrier in mammals, especially in the case of increased intestinal permeability, disseminate via the bloodstream, locate in host tissues, and internalize with cells. Circulating PG goes mainly to the kidneys, but it also appears in the spleen, thymus, fat, liver, lungs, and even the brain [48, 49]. The concentrations of PG achieved in host tissues are very low, ranging from 10–100 ng/g in organs to 20 ng/mL in blood. However, the effects observed are important for host physiology, including immunomodulatory activity through interactions with host cell receptors such as NOD‐1 or TLR2. Bacterial PG activation of nuclear factor kappa‐light‐chain‐enhancer of activated B cells (NF‐κB), p38, and extracellular‐signal‐regulated kinase (Erk) increases production of proinflammatory cytokines such as macrophage inflammatory protein 2 (MIP‐2) and IL‐8, also interferon type I (IFN I) and β‐defensins. PG activates the complement cascade on the lectin pathway, may induce apoptosis and autophagy, and also possesses pyrogenic and somnogenic properties after recognition by serotonin receptors [48]. SpA has been detected in the skin lesions and fluids of animal models of MRSA infections and patients infected with *S. aureus*, including those suffering from atopic dermatitis [50, 51]. The concentration of SpA in host tissues has not been estimated, but its inhibitory effect on adaptive immune response through binding of Fc regions of IgG and B cell receptors (BCRs), as well as innate immune response by blocking IgG hexamerization and thus complement activation and induction of leukocyte necrosis is emphasized [51–53]. In our study, *S. aureus* ATCC 43300 (MRSA) planktonic and biofilm cultures were first exposed for 24 h to *V. opulus* extracts, and for comparison also to ChA as the component of all tested extracts and VAN as an antibiotic commonly used against MRSA. The spontaneous release of PG and SpA from planktonic and biofilm *S. aureus* cultures was checked in control samples: K1—bacteria in medium alone (control for all tested samples excluding these treated with ChA) and K2—bacteria in medium containing 0.5% ethanol (Et‐OH) being ChA solvent. The results of the PG release are presented in Table 1.

**Table 1:** The effect of *V. opulus* L. extracts on peptidoglycan (PG) release from *S. aureus* ATCC 43300 growing in planktonic and biofilm form.

Experimental groups	PG concentration (ng/mL)	
	Planktonic	Biofilm
K1	47.97 ± 24.94	18.83 ± 8.68
K2	30.93 ± 4.22	22.81 ± 11.07
VBw	66.58 ± 23.60	31.28 ± 9.68
VFw	62.03 ± 30.74	28.04 ± 9.49
VFe	67.97 ± 21.05	27.46 ± 9.88
ChA	33.83 ± 26.80	22.31 ± 9.08
VAN	54.07 ± 23.12	24.80 ± 7.12

*Note:* PG concentrations are expressed as the mean values ± SD (*n* = 4). VF, *V. opulus* fruit extract; VB, *V. opulus* bark extract; e/w, ethanolic/water extract; ChA, chlorogenic acid (100 µg/mL); VAN, vancomycin (½ MIC); K1, control bacteria in medium alone, K2, control bacteria in medium containing 0.5% Et‐OH.

The PG and SpA were detected in all tested and control samples, although the level of SpA was low, ranging from 5.22 ± 3.60 to 8.23 ± 1.16 ng/mL and from 4.45 ± 2.41 to 5.90 ± 0.42 ng/mL for planktonic and biofilm *S. aureus* cultures, respectively. Moreover, it did not differ from SpA concentrations released spontaneously in control samples (from 5.23 ± 2.21 to 6.34 ± 2.82 ng/mL). While the PG content reached even 68 ng/mL and was higher by 12.5%–41.7% in planktonic cultures and 31.7%–66.1% in biofilm cultures than in appropriate controls (spontaneous PG release). Interestingly, PG release from bacteria exposed to *V. opulus* extracts was even higher than from those exposed to the antibiotic used at ½ MIC (Table 1). Obtained concentrations of PG in staphylococcal culture supernatants were similar to PG levels achieved in host tissues, ranging from 10 to 100 ng/g or mL, exerting biological effects [48]. To summarize, plant extracts may act as trigger factors for the gentle release of bioactive bacterial cell components.

### 3.2. *S. aureus* Culture Supernatants Do Not Alter the Surface Expression of CD Polarization Markers on the THP‐1‐Derived Macrophage In Vitro

**Figure 1:**
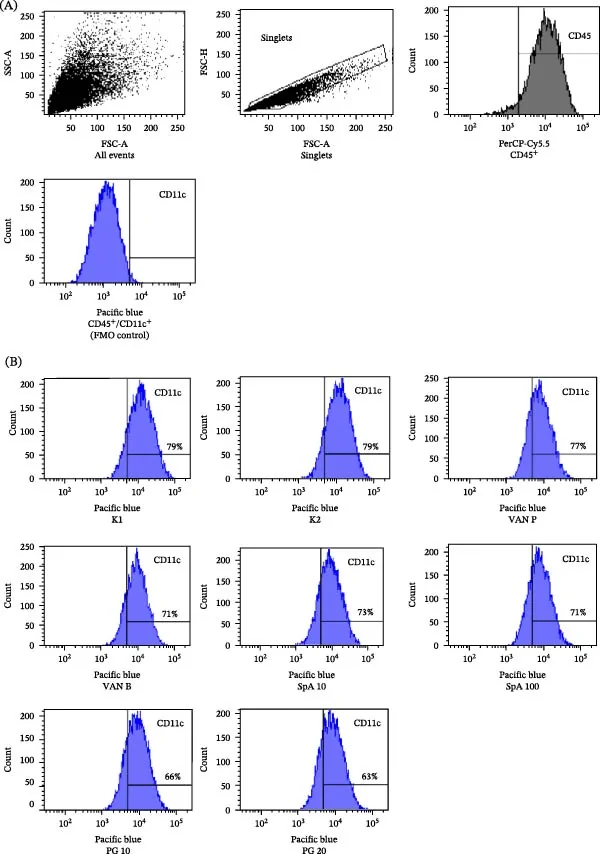
Representative flow cytometry dot‐plots and histograms for the CD marker expression on THP‐1‐derived macrophages exposed (24 h) to different stimuli. (A) Gating scheme for the measurement of CD11c expression. Light scatter profile for the cells based on forward scatter (FSC‐A) and side scatter (SSC‐A). Singlet gating based on FSC‐H vs. FSC‐A (the region was set to discriminate cell doublets). Macrophages were then gated based on CD45 expression (CD45^+^ objects). CD11c expression on CD45^+^ objects was gated against FMO (fluorescence minus one). (B) Flow cytometry histograms of CD11c expression on the macrophages (CD45^+^ objects): K1, control cells in culture medium alone (control for the cells treated with bacterial cell wall components); K2, control cells in culture medium containing growth medium for bacteria, TSB/Glu (control for the cells treated with *S. aureus* culture supernatants); VAN P/B, vancomycin planktonic/biofilm supernatants; SpA 10/100, staphylococcal protein A (10/100 µg/mL); PG 10/20, peptidoglycan (10/20 µg/mL).

**Figure 2:**
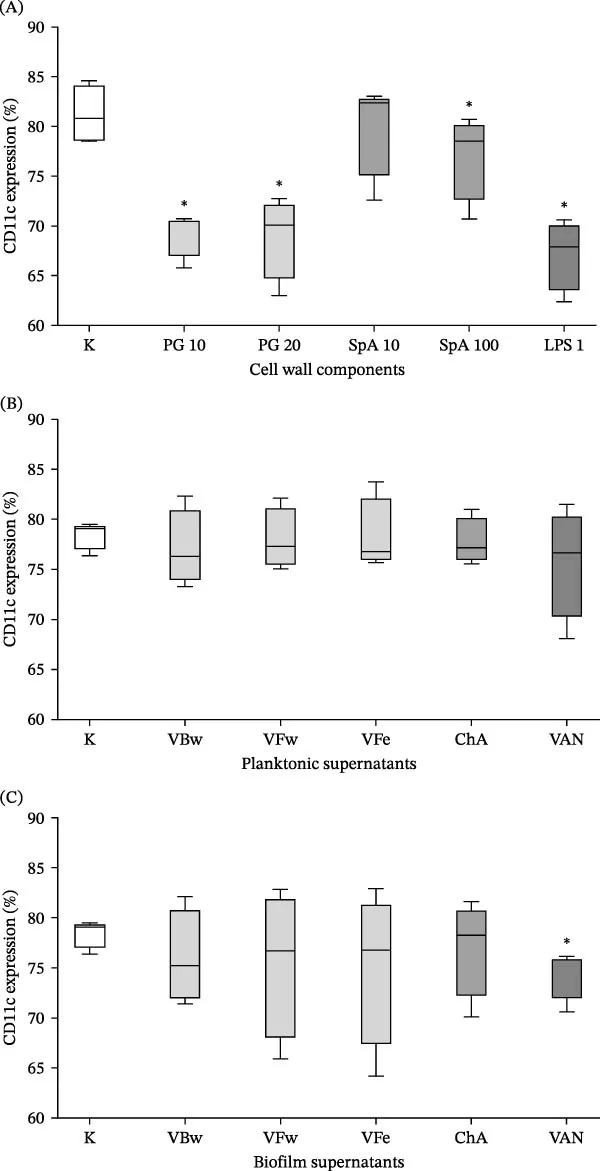
CD11c surface expression on THP‐1‐derived macrophages (CD45^+^ cells) exposed (24 h) to different stimuli. (A) Bacterial cell wall components: PG, *S. aureus* peptidoglycan (10 and 20 µg/mL); SpA, *S. aureus* protein A (10 and 100 µg/mL); LPS, *E. coli* LPS (1 µg/mL). (B) The supernatants from *S. aureus* ATCC 43300 planktonic cultures. (C) The supernatants from *S. aureus* ATCC 43300 biofilm cultures. The supernatants were obtained after staphylococcal cultures treatment with: VBw, *V. opulus* bark water extract; VFw, *V. opulus* fruit water extract; VFe, *V. opulus* fruit ethanolic extract (all at 10%, v/v); ChA, chlorogenic acid (100 µg/mL); VAN, vancomycin (½ MIC).  ^∗^ *p* ≤ 0.05 statistically significant differences compared to appropriate controls (K): unexposed cells in culture medium alone (control for the cells exposed to bacterial cell wall components) or in culture medium containing 10% (v/v) growth medium for bacteria (control for the cells exposed to *S. aureus* culture supernatants).

**Figure 3:**
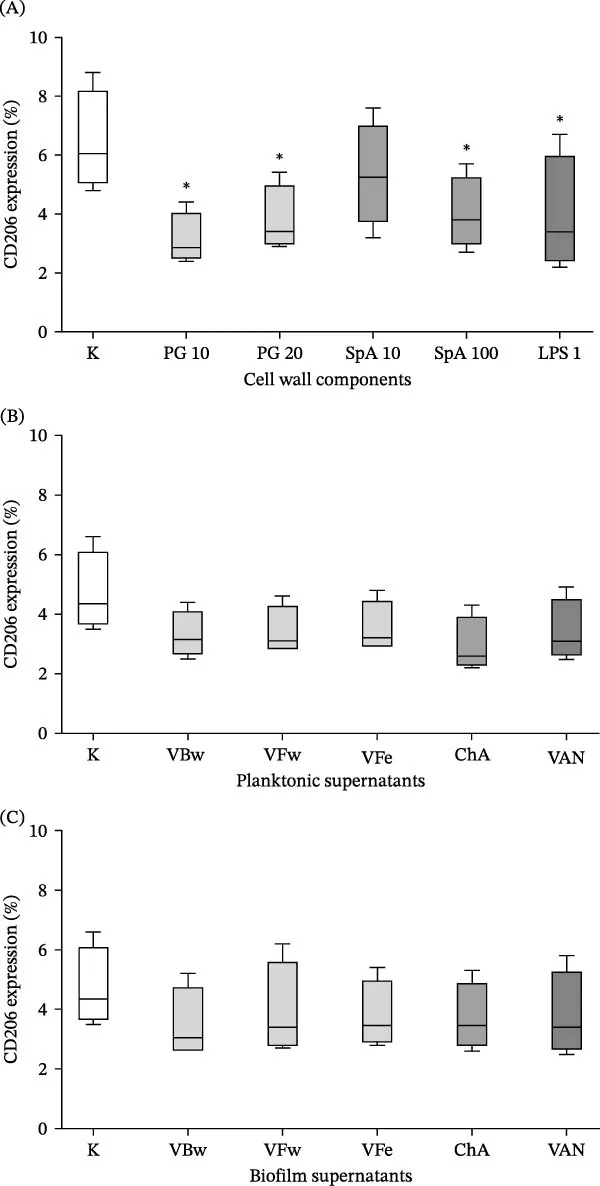
CD206 surface expression on THP‐1‐derived macrophages (CD45^+^ cells) exposed (24 h) to different stimuli. (A) Bacterial cell wall components: PG, *S. aureus* peptidoglycan (10 and 20 µg/mL); SpA, *S. aureus* protein A (10 and 100 µg/mL); LPS, *E. coli* LPS (1 µg/mL). (B) The supernatants from *S. aureus* ATCC 43300 planktonic cultures. (C) The supernatants from *S. aureus* ATCC 43300 biofilm cultures. The supernatants were obtained after staphylococcal cultures treatment with: VBw, *V. opulus* bark water extract; VFw, *V. opulus* fruit water extract; VFe, *V. opulus* fruit ethanolic extract (all at 10%, v/v); ChA, chlorogenic acid (100 µg/mL); VAN, vancomycin (½ MIC).  ^∗^ *p* ≤ 0.05 statistically significant differences compared to appropriate controls (K): unexposed cells in culture medium alone (control for the cells exposed to bacterial cell wall components) or in culture medium containing 10% (v/v) growth medium for bacteria (control for the cells exposed to *S. aureus* culture supernatants).

These experiments aimed to analyze the potential of plant‐origin dietary supplements and the physiological microbiota to modulate innate immune cell polarization. The impact of *S. aureus* planktonic and biofilm culture supernatants after bacteria exposure to *V. opulus* extracts, ChA, and Van on the CD marker surface expression was tested in vitro on THP‐1‐derived CD45^+^ cells recognized as macrophages. The cells were also exposed to bacterial cell wall components for comparison. CD11c as a marker of M1 and CD206 as a marker of M2 were chosen based on literature data [54–56] to assess the potential polarization of the macrophages. The results are presented in Figures 1–3.

The expression of CD11c on unexposed (control) cells was very high – ~80% of CD45^+^ cells expressed CD11c on the surface (Figure 2A–C), suggesting that THP‐1 cell polarization to the M1‐like phenotype occurred under stimulation with PMA alone. PMA is not a polarizing agent in the classical sense; it is used only to differentiate THP‐1 monocytes into macrophages with an M0 phenotype (neutral, nonpolarized), and PMA‐treating cells are followed by a resting period. Moreover, PMA enhances the features of mature macrophages while preserving response plasticity [57]. However, the observed high baseline expression of CD11c creates a potential “ceiling effect” limiting the detection of further changes in the M1 marker exposure and making conclusions about cell polarization. In our study, subsequent exposure of the PMA‐treated cells to *S. aureus* planktonic or biofilm supernatants did not notably decrease the CD11c expression (Figure 2B,C). Only the cells exposed to the supernatant of the *S. aureus* biofilm treated with VAN exhibited significantly lower expression of CD11c, with a 4% decline compared to the control cells (Figure 2C). Similarly, pure bacterial components such as PG and LPS used at much higher concentrations (10 and 20 μg/mL of pure PG vs. 62–66 ng/mL PG in bacterial supernatants) significantly decreased CD11c exposition (Figure 2A).

At the same time, we did not observe any increase in CD206 exposure, which would indicate changes in macrophage polarization. The cells exposed to pure bacterial cell wall components (Figure 3A) and *S. aureus* planktonic and biofilm culture supernatants (Figure 3B,C) also expressed a lower level of CD206. While differences from the control cells were significant only for pure bacterial components (Figure 3A). The PG used at 10–20 μg/mL and LPS at 1 μg/mL did not alter the viability of the cells. Still, it is possible that these components violated the composition and functionality of their cell membranes, leading to a disturbance in CD exposition.

Assessment of phenotypic changes in the THP‐1 cells differentiated with PMA (24 h) followed by a 72‐h resting period should take into account that PMA induces a high baseline CD11c expression in the M0 phenotype. It may cause a “ceiling effect” and limit the detection of subtle changes during cell polarization [58]. Under these conditions, interpreting the cell polarization solely on the basis of the surface marker’s expression (CD11c and CD206) is insufficient. In this study, we also tested cytokine responses and effector functions of THP‐1‐derived macrophages exposed to the supernatants from planktonic and biofilm *S. aureus* cultures pretreated with plant extracts or to pure bacterial cell wall components, which may be more sensitive indicators of the cell response than changes in the surface CD marker’s expression.

### 3.3. *S. aureus* Culture Supernatants and Bacterial Cell Wall Components Significantly Increase the Cytokine Production by THP‐1‐Derived Macrophages

**Figure 4:**
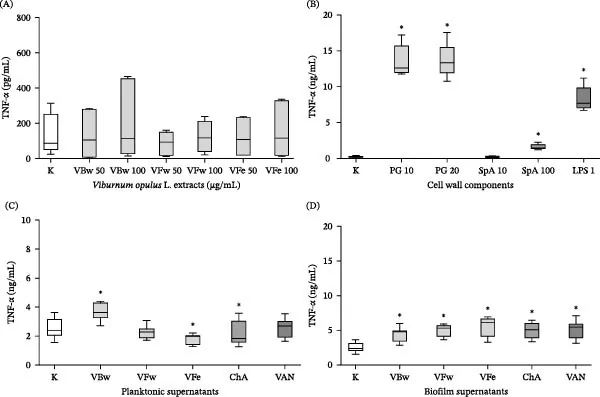
TNF‐α production by THP‐1‐derived macrophages exposed (24 h) to different stimuli. (A) *Viburnum opulus* L. extracts: VBw, *V. opulus* bark water extract; VFw, *V. opulus* fruit water extract; VFe, *V. opulus* fruit ethanolic extract (all at 50 and 100 μg/mL). (B) Bacterial cell wall components: PG, *S. aureus* peptidoglycan (10 and 20 µg/mL); SpA, *S. aureus* protein A (10 and 100 µg/mL); LPS, *E. coli* LPS (1 µg/mL). (C) The supernatants from *S. aureus* ATCC 43300 planktonic cultures. (D) The supernatants from *S. aureus* ATCC 43300 biofilm cultures. The supernatants were obtained after staphylococcal cultures treatment with: VBw, *V. opulus* bark water extract; VFw, *V. opulus* fruit water extract; VFe, *V. opulus* fruit ethanolic extract (all at 10%, v/v); ChA, chlorogenic acid (100 µg/mL); VAN, vancomycin (½ MIC).  ^∗^ *p* ≤ 0.05 statistically significant differences compared to appropriate controls (K): unexposed cells in culture medium alone (control for the cells exposed to *V. opulus* extracts and bacterial cell wall components) or in culture medium containing 10% (v/v) growth medium for bacteria (control for the cells exposed to *S. aureus* culture supernatants).

**Figure 5:**
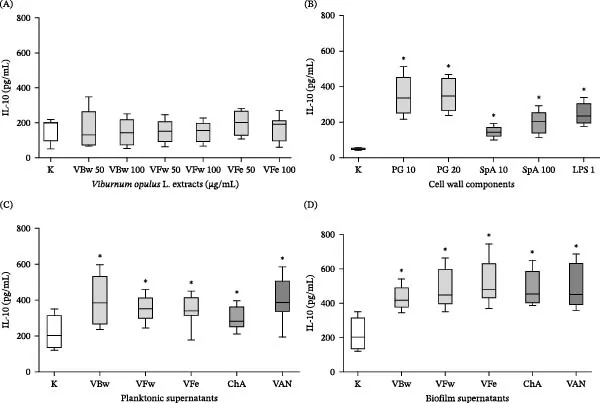
IL‐10 production by THP‐1‐derived macrophages exposed (24 h) to different stimuli. (A) *Viburnum opulus* L. extracts: VBw, *V. opulus* bark water extract; VFw, *V. opulus* fruit water extract; VFe, *V. opulus* fruit ethanolic extract (all at 50 and 100 μg/mL). (B) Bacterial cell wall components: PG, *S. aureus* peptidoglycan (10 and 20 µg/mL); SpA, *S. aureus* protein A (10 and 100 µg/mL); LPS, *E. coli* LPS (1 µg/mL). (C) The supernatants from *S. aureus* ATCC 43300 planktonic cultures. (D) The supernatants from *S. aureus* ATCC 43300 biofilm cultures. The supernatants were obtained after staphylococcal cultures treatment with: VBw, *V. opulus* bark water extract; VFw, *V. opulus* fruit water extract; VFe, *V. opulus* fruit ethanolic extract (all at 10%, v/v); ChA, chlorogenic acid (100 µg/mL); VAN, vancomycin (½ MIC).  ^∗^ *p* ≤ 0.05 statistically significant differences compared to appropriate controls (K): unexposed cells in culture medium alone (control for the cells exposed to *V. opulus* extracts and bacterial cell wall components) or in culture medium containing 10% (*v/v*) growth medium for bacteria (control for the cells exposed to *S. aureus* culture supernatants).

The potential of plant extracts and microbiota models to modulate the secretory activity of innate immune cells was tested in vitro on THP‐1‐derived macrophages exposed to *S. aureus* culture supernatants. The cells were also comparatively exposed to *V. opulus* L. extracts alone and bacterial cell wall components. Production of proinflammatory and anti‐inflammatory representative cytokines, such as TNF‐α and IL‐10, respectively, was assessed by the ELISA method. The results are presented in Figures 4 and 5.

TNF‐α production by THP‐1‐derived macrophages increased significantly after their exposure to bacterial cell wall components (Figure 4B) and *S. aureus* biofilm culture supernatants (Figure 4D). In the case of *S. aureus* planktonic culture supernatants, a significant increase of TNF‐α production was noted only for the macrophages exposed to VBw (bacteria treated with VBw extract) (Figure 4C). The observed boost of TNF‐α secretion for *S. aureus* culture supernatants was between 1.8 and 2.2 times. The most remarkable secretion spike of this cytokine was exhibited by THP‐1‐derived macrophages exposed to PG (61–62 fold increase), confirming the proinflammatory effects of *S. aureus* PG. IL‐10 production increased significantly in the macrophages exposed to bacterial cell wall components and *S. aureus* culture supernatants (Figure 5B–D). Similarly, the most intense enhancement was demonstrated for THP‐1‐derived macrophages exposed to PG (seven times, Figure 5B). The concentration of IL‐10 increased up to 2.4 times and 1.8 times in the case of the cell exposure to biofilm and planktonic culture supernatants, respectively. To exclude the influence of plant extracts themselves, we also assessed the direct impact of *V. opulus* extracts used at concentrations of 50 and 100 μg/mL on THP‐1‐derived cells (Figures 4A and 5A for TNF‐α and IL‐10, respectively). TNF‐α production was at a very low level, achieving 0.0025–0.464 ng/mL for VB extracts and 0.0106–0.335 ng/mL for VF extracts. Similarly, IL‐10 production achieved was only 0.0529–0.347 ng/mL for VB extracts and 0.0593–0.281 ng/mL for VF extracts. We did not notice any statistical differences in TNF‐α and IL‐10 production between tested cells (extract‐treated) and control (untreated) cells. What’s more, in the present study, the final concentration of plant extracts during the cell exposure to microbial supernatants was at least 10 times lower than tested directly (about 10 μg/mL), if bacteria were treated with *V. opulus* extracts used at 100 μg/mL, and then the THP‐1‐derived cells were exposed to microbial supernatants added at a concentration of 10% (v/v). Taking this into account, the influence of plant extracts themselves on cytokine production seems to be excluded.

The potent impact of staphylococcal culture supernatants and pure bacterial cell wall components on cytokine secretion is observed. Generally, TNF‐α and IL‐10 were overproduced by the exposed macrophages compared to control cells. Interestingly, Zielinski et al. [59] demonstrated that human blood lymphocytes primed *in vitro* with inactivated *S. aureus* developed predominantly Th1/Th17 cells, which were capable of IL‐10 production upon restimulation. Moreover, such a response seemed to be pathogen‐specific since it was not observed in *Candida albicans*‐induced cells [59]. We cannot fully confirm this thesis because of the different study setup (we evaluated the direct response of the cells, not their response upon restimulation), and LPS of *E. coli* used in our study also increased IL‐10 secretion. Nevertheless, it is an interesting analogy, and the effect of LPS that we noticed was weaker than that of *S. aureus* PG. The profile of cytokines produced may depend on the qualitative composition of culture supernatants used as cell stimulators and the concentration of the active components that they contain. Choi et al. [60] demonstrated that THP‐1‐derived macrophages exposed to staphylococcal enterotoxin B (SEB) used at 2 *vs*. 20 μg/mL showed diverse cytokine production (RANTES, IL‐8, CXCL11, and IFN‐β *vs*. additionally produced MCP‐1, MIP‐1, CCL20, 22, 24, IP‐10, TNF‐α, and sICAM‐1). It may explain the differences observed in our study in TNF‐α production between the cells exposed to different stimuli, bearing in mind that the concentration gradient of their active components was arranged as follows: pure bacterial cell wall components > planktonic supernatants > biofilm supernatants.

### 3.4. *S. aureus* Culture Supernatants Do Not Alter the Phagocytic Function of THP‐1‐Derived Macrophages

**Figure 6:**
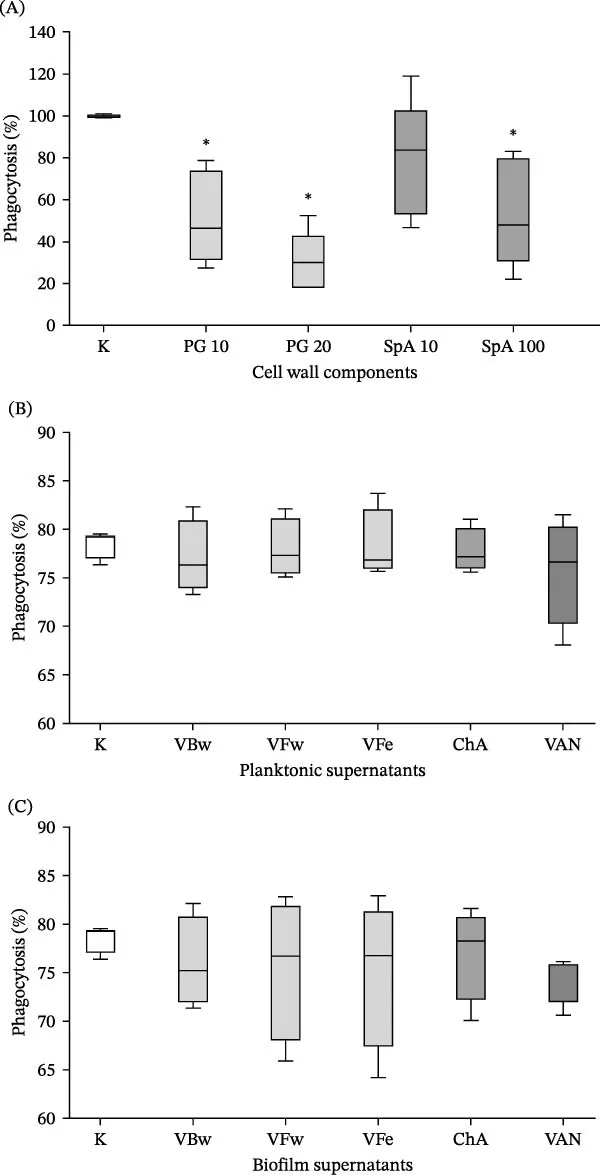
Uptake of *S. aureus* ATCC 43300 by THP‐1‐derived macrophages exposed (24 h) to different stimuli. (A) Bacterial cell wall components: PG, *S. aureus* peptidoglycan (10 and 20 µg/mL); SpA, *S. aureus* protein A (10 and 100 µg/mL). (B) The supernatants from *S. aureus* ATCC 43300 planktonic cultures. (C) The supernatants from *S. aureus* ATCC 43300 biofilm cultures. The supernatants were obtained after staphylococcal cultures treatment with: VBw, *V. opulus* bark water extract; VFw, *V. opulus* fruit water extract; VFe, *V. opulus* fruit ethanolic extract (all at 10%, v/v); ChA, chlorogenic acid (100 µg/mL); VAN, vancomycin (½ MIC).  ^∗^ *p* ≤ 0.05 statistically significant differences compared to appropriate controls (K): unexposed cells in culture medium alone (control for the cells exposed to bacterial cell wall components) or in culture medium containing 10% (v/v) growth medium for bacteria (control for the cells exposed to *S. aureus* culture supernatants).

**Figure 7:**
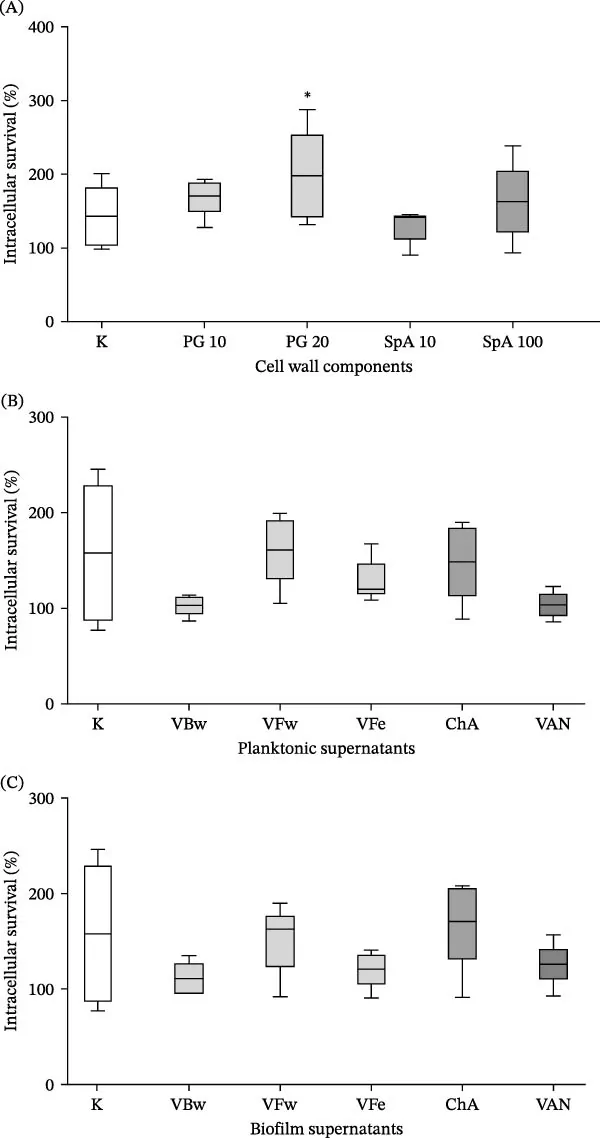
Survival of *S. aureus* ATCC 43300 inside the THP‐1‐derived macrophages exposed (24 h) to different stimuli. (A) Bacterial cell wall components: PG, *S. aureus* peptidoglycan (10 and 20 µg/mL); SpA, *S. aureus* protein A (10 and 100 µg/mL). (B) The supernatants from *S. aureus* ATCC 43300 planktonic cultures. (C) The supernatants from *S. aureus* ATCC 43300 biofilm cultures. The supernatants were obtained after staphylococcal cultures treatment with: VBw, *V. opulus* bark water extract; VFw, *V. opulus* fruit water extract; VFe, *V. opulus* fruit ethanolic extract (all at 10%, v/v); ChA, chlorogenic acid (100 µg/mL); VAN, vancomycin (½ MIC).  ^∗^ *p* ≤ 0.05 statistically significant differences compared to appropriate controls (K): unexposed cells in culture medium alone (control for the cells exposed to bacterial cell wall components) or in culture medium containing 10% (v/v) growth medium for bacteria (control for the cells exposed to *S. aureus* culture supernatants).

To evaluate the potential of plant‐based dietary supplements and host microbiota to modulate the innate immune cell phagocytic functions, the THP‐1‐derived macrophages were exposed to *S. aureus* culture supernatants, and their ability to engulf and intracellularly kill staphylococci was tested. The cells were also comparatively exposed to bacterial cell wall components. The effectiveness of staphylococcal engulfment (% of phagocytosis) and intracellular killing expressed as a percentage of bacteria that survived inside the cells is presented in Figures 6 and 7, respectively.

A statistically significant decrease in *S. aureus* ATCC 43300 uptake by THP‐1‐derived macrophages exposed to bacterial cell wall components was observed (Figure 6A). PG used at 20 µg/mL caused the most significant decrease, reducing the engulfment of bacteria by 69.6% (*p* ≤ 0.001). A similar effect was observed after cell exposure to SpA used at 100 µg/mL (decline up to 47%). While exposure of the macrophages to *S. aureus* culture supernatants (both planktonic and biofilm) after bacterial treatment with *V. opulus* extracts, ChA, or VAN did not exert a notable impact on *S. aureus* engulfment (Figure 6B,C).

First of all, we demonstrated that *S. aureus* ATCC 43300 survived and multiplicated inside THP‐1‐derived macrophages during 24 h after internalization. The number of alive bacteria increased from about 43% to 58% in control cells (K) compared to the number of initially engulfed bacteria, which was deemed 100% (Figure 7). Flannagan et al. [61] also showed the growth and replication of *S. aureus* inside fully acidified RAW 264.7 macrophage phagolysosomes, which required the expression of the bacterial GraXRS regulatory system. Interestingly, in our study, the rate of staphylococcal multiplication differed between cells exposed to different stimuli. Staphylococcal intracellular survival intensified significantly in the cells exposed to pure PG (by 25.5% and 54.8% after exposure to PG at 10 and 20 µg/mL, respectively) compared to that in control cells (Figure 7A). While *S. aureus* culture supernatants (both planktonic and biofilm) obtained after bacterial treatment with *V. opulus* extracts, ChA, or VAN limited the multiplication rate of *S. aureus* during the next 24 h after uptake inside exposed THP‐1 macrophages compared to the control cells (Figure 7B,C). Although the differences were not statistically significant, it is worth noting the increased bacteriostatic activity of macrophages after exposure to staphylococcal culture supernatants. Interestingly, the greatest inhibition of bacterial multiplication was observed in cells exposed to the supernatants of *S. aureus* pretreated with the VBw extract. Exactly the same ones that most effectively stimulated the cells to produce TNF‐α (Figure 4B,C). Those two effects of VBw‐treated supernatants on THP‐1 macrophages may be connected to each other. TNF‐α is a potent stimulator of macrophage activity, including the inhibition of growth and intracellular killing of microorganisms.

Gidon et al. [62] demonstrated that TNF‐α together with IL‐6 controlled the intracellular growth of *Mycobacterium avium* in human primary macrophages through activation of the transcription factor IRF1 (interferon regulatory factor 1) and expression of IRG1, a mitochondrial enzyme that produces itaconate, the antimicrobial metabolite. Thus, with high probability, we can point to increased expression of TNF‐α as one of the mechanisms of “trained innate immunity” realized by the contact of immune cells with bioactive staphylococcal components released from these bacteria.

## 4. Conclusions

The study demonstrated that *V. opulus* extracts used at a low concentration of 100 µg/mL released biologically active components from *S. aureus* cells growing both in planktonic and biofilm form. Then, these factors targeted innate immunity against staphylococci in an *in vitro* model by enhancing cytokine production (in particular regulatory IL‐10) and reducing intracellular multiplication of *S. aureus*. However, such aspects of the macrophage activity as polarization assessed by the CD marker surface exposition and the first step of phagocytosis (engulfment of microorganisms) did not change under the microbial supernatant treatment. The lack of changes in the surface marker exposition may be a limitation of the model (PMA‐differentiated THP‐1) rather than the phenomenon itself. Some data suggests that to induce a trained phenotype of macrophages, the cooperation of other immune cells, such as T lymphocytes, is required [41]. Thus, further studies in more complex systems involving different types of cells cooperating together and a mixed population of microorganisms better reflecting the resident microbiota would be needed. Nevertheless, we may consider using in the future plant extracts as triggers to release immunomodulatory components from their own host microbiota.

## Funding

The studies were supported by the University of Lodz Doctoral Research Grant for U. Wójcik‐Bojek.

## Conflicts of Interest

The authors declare no conflicts of interest.

## Data Availability

All data generated or analyzed during this study are included in this article. The datasets are available from Beata Sadowska (beata.sadowska@biol.uni.lodz.pl) upon reasonable request.
